# Development of a low-cost IoT system based on LoRaWAN for monitoring variables related to electrical energy consumption in low voltage networks

**DOI:** 10.1016/j.ohx.2022.e00330

**Published:** 2022-06-18

**Authors:** Nelson E. Guevara, Yamir H. Bolaños, Juan P. Diago, Juan M. Segura

**Affiliations:** a Faculty of Electronic Engineering, Corporación Universitaria Autónoma del Cauca, Colombia; b Faculty of Engineering, Fundación Universitaria de Popayán, Colombia; c Maintenance Area, Industria Licorera del Cauca, Colombia

**Keywords:** IoT system, Energy consumption, Energy meter, LoRa, LoRaWAN, Local web server, Web application

## Abstract

Energy efficiency is an issue that is currently gaining relevance, high electricity demands worldwide generate a negative impact on the planet caused by the natural depletion of resources associated with production processes. In this regard, the technologies associated with the Internet of Things (IoT) are considered as a tool to optimize processes and resources through the monitoring of variables. In this context, this work proposes a low-cost electronic system with IoT architecture used in the monitoring of electrical variables, this becomes a support tool in the estimation of energy consumption in internal distribution electrical circuits of homes or small industries. This device generates information to recognize consumption patterns and load balances per electrical phase, contains two hardware modules and a software user interface. The first is an electronic node that includes a high-performance polyphase meter based on the Atmel M90E32AS chip, which is controlled by an ESP32 chip, for wireless communication is used a Radio Frequency (RF) module in the 915 MHz band and LoRa protocol based on the Semtech SX1278 transceiver, this node is able to measure and transmit variables such as current, voltage, active energy, reactive energy, power factor and other electrical variables in circuits of up to three phases. For the study, a calibration process was carried out in an accredited laboratory (Metrex S.A. in Colombia), then tests were performed by monitoring a three-phase 110V electrical circuit in a small factory, with the information generated it was possible to identify consumption patterns over a period of seven consecutive days, important data such as times when energy is wasted due to improper use of loads connected to the network, electric stoves, computer equipment turned on during non-working hours are examples of the results obtained.

## Specifications table

**Table:** 

Hardware name	*IEMS (IoT Electrical Monitoring System)*
Subject area	*Engineering*
Hardware type	*Measuring* *Field measurements and sensors* *Electronic engineering*
Open-Source License	*CC BY 4.0*
Cost of Hardware	*$130 USD*
Source File Repository	https://doi.org/10.17632/bkhrks6x3m.2

## Hardware in context

Currently there is high interest in the optimization of energy expenditure considering the world has limited non-renewable resources, the electricity sector is one of the most important given the massive use of electricity, so efforts are constantly made to use technology to optimize processes, the change of traditional lamps to LED type, generation of efficient power supplies, are some examples, however there are also other actions such as monitoring of consumption variables, to have updated information in short periods of time can detect consumption habits and the use of equipment with inefficient consumption.

The present work proposes the development of a low-cost system for monitoring electrical variables in distribution circuits in low voltage networks, Some research papers can be cited oriented to the generation patterns and characteristics of energy consumption [1], [2]. In [3] the authors present a platform called IoTEP (IoT Energy Platform) that manages data for energy analysis using electrical variables such as active power and reactive power, this was evaluated in a real case that included data from internal circuits of the University of Murcia, Spain, the authors claim that the platform has good performance for being flexible, robust and promote the reduction of energy expenditure. Although the system in [3] was validated and its contribution is significant, it lacks information regarding the electrical metering device used; similar case in [4], [5]. Another work described in [6] presents an IoT architecture based on the LoRaWAN communication protocol to form an AMI (Advanced Metering Infrastructure) network, the authors discuss the benefits of LoRa technology as a promising technology for LPWAN (Low Power Wide Area Network) networks, other works explore the use of RF Zigbee technologies to form short range wireless networks, where several points are required to cover a large area which increases integration costs to scale the system [7], [8], [9].

From the present review, although there are important research contributions, many of them do not clearly describe how to replicate the device; case of [3], [4], [5], [6], or use short-range wireless devices; case [8], on the other hand, there are similar equipment with high costs, and they are used in certification process or specialized requirements. In that sense, the proposed system apart from proposing an intuitive and open source IoT interface highlights the use of LoRa technology for long-range wireless data transmission and connection to a web platform, which allows analyzing the behavior of different electrical variables such as: voltage, current, active power, reactive power, power factor, frequency, among others, in a three-phase network. Consequently, the proposed device can be cataloged as low cost, replicable, portable and open access to use for a basic monitoring.

## Hardware description

**Fig. 1:**
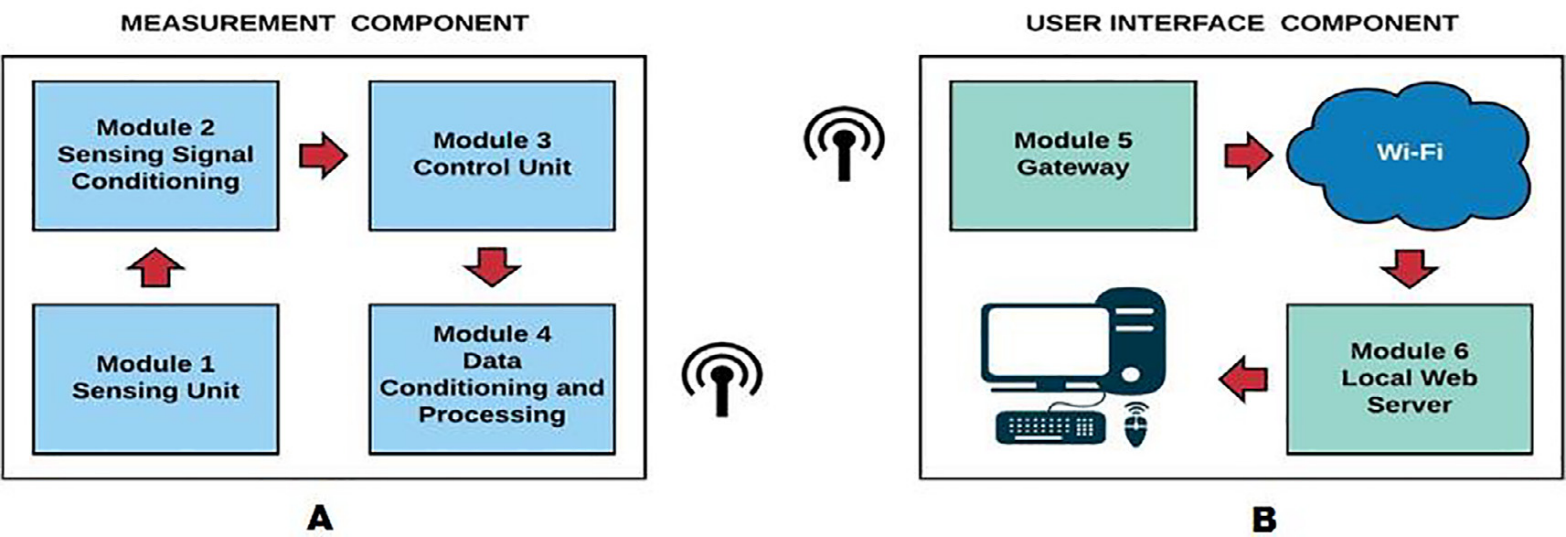
System architecture.

The IoT Electrical Monitoring System (IEMS), proposed in the current paper, is based on a modular architecture as shown in Fig. 1, in A on the left is the first component (hardware device) for the measurement of energy variables called energy meter and consists of four internal modules (Sensing unit, Conditioning of sensing signals, Control unit and Information preprocessing), which make a connection with the module number 5 (LoRa gateway) of the B part. The communication between them is done wirelessly through LoRa protocol at a frequency of 915 MHz [10] and with a maximum distance of 5 Km in line of sight, the LoRa gateway communicates to the cloud through a WiFi connection to send the collected data to the monitoring software platform. A block diagram summarizing the described system is presented below.

To facilitate the understanding of the system and the modules in each component mentioned above, they are briefly explained below:

• **Module 1:** Composed of three YHDC SCT016S [11], current transducers, which specifically measure maximum voltages of 655V and maximum currents of 65A.
• **Module 2:** An Atmel M90E32AS chip [12], which is a polyphase measurement integrated circuit capable of performing the calculation of the main electrical variables, conventionally measured such as: voltage, current, active energy, reactive energy, apparent energy, active power, reactive power, apparent power, power factor, frequency, phase angle and temperature in single-phase, two-phase or three-phase circuits.
• **Module 3:** An ESP32 VROOM microcontroller [13], which is a low-cost element and low power consumption, which is in charge of obtaining the readings made by the meter chip of the electrical variables.
• **Module 4 and 5:** Two Heltec ESP32 LoRa development boards [14], which enable communication and operation via classic IoT elements with Bluetooth, Wi-Fi and LoRa functions.

**Fig. 2:**
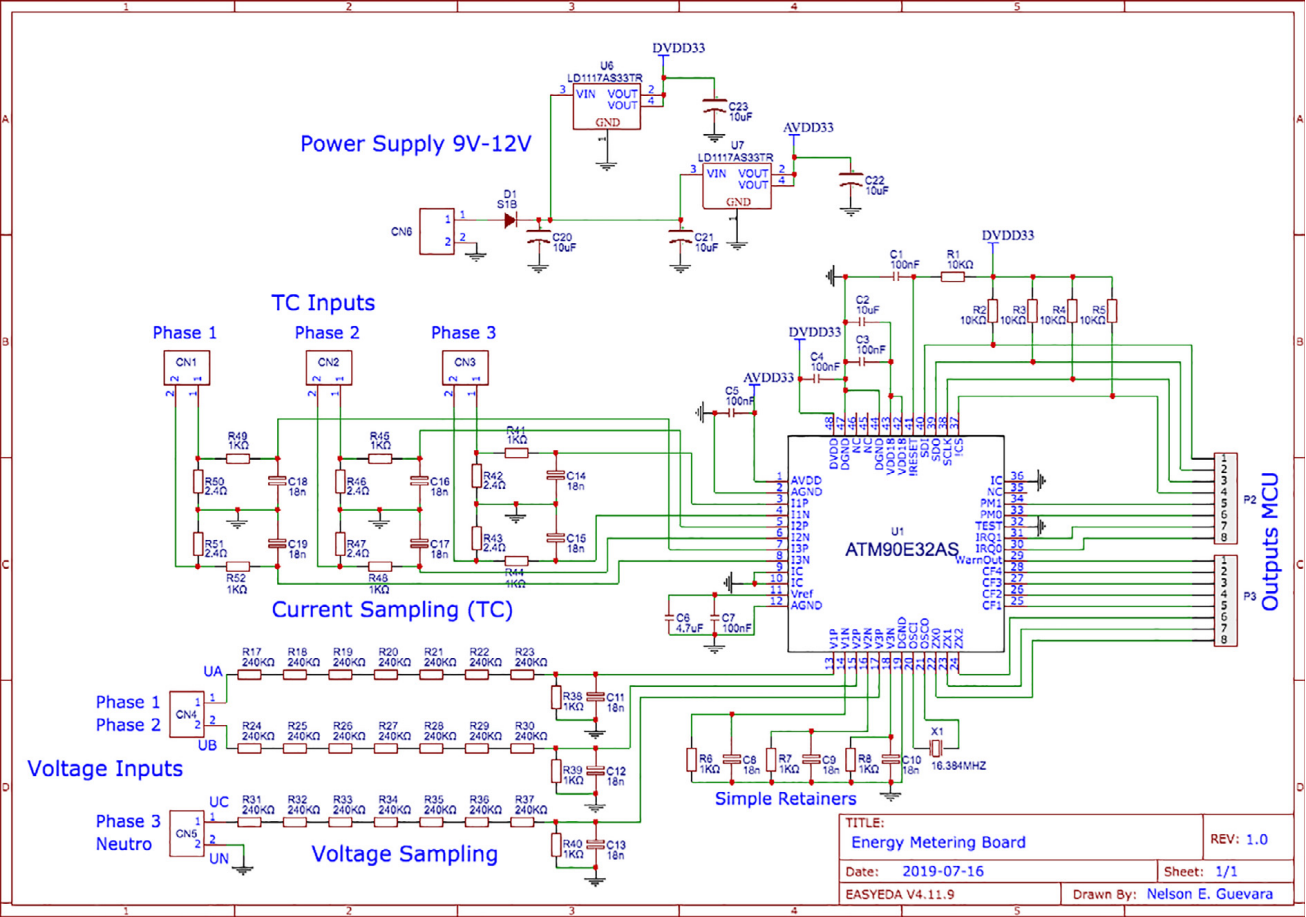
Schematic diagram reference for energy meter.

During the implementation phase, an interconnection schematic diagram was done based on Atmel-M90E32AS Application Note [15], shown in Fig. 2, which corresponds to the energy meter device equipped with the Atmel M90E32AS chip.

**Fig. 3:**
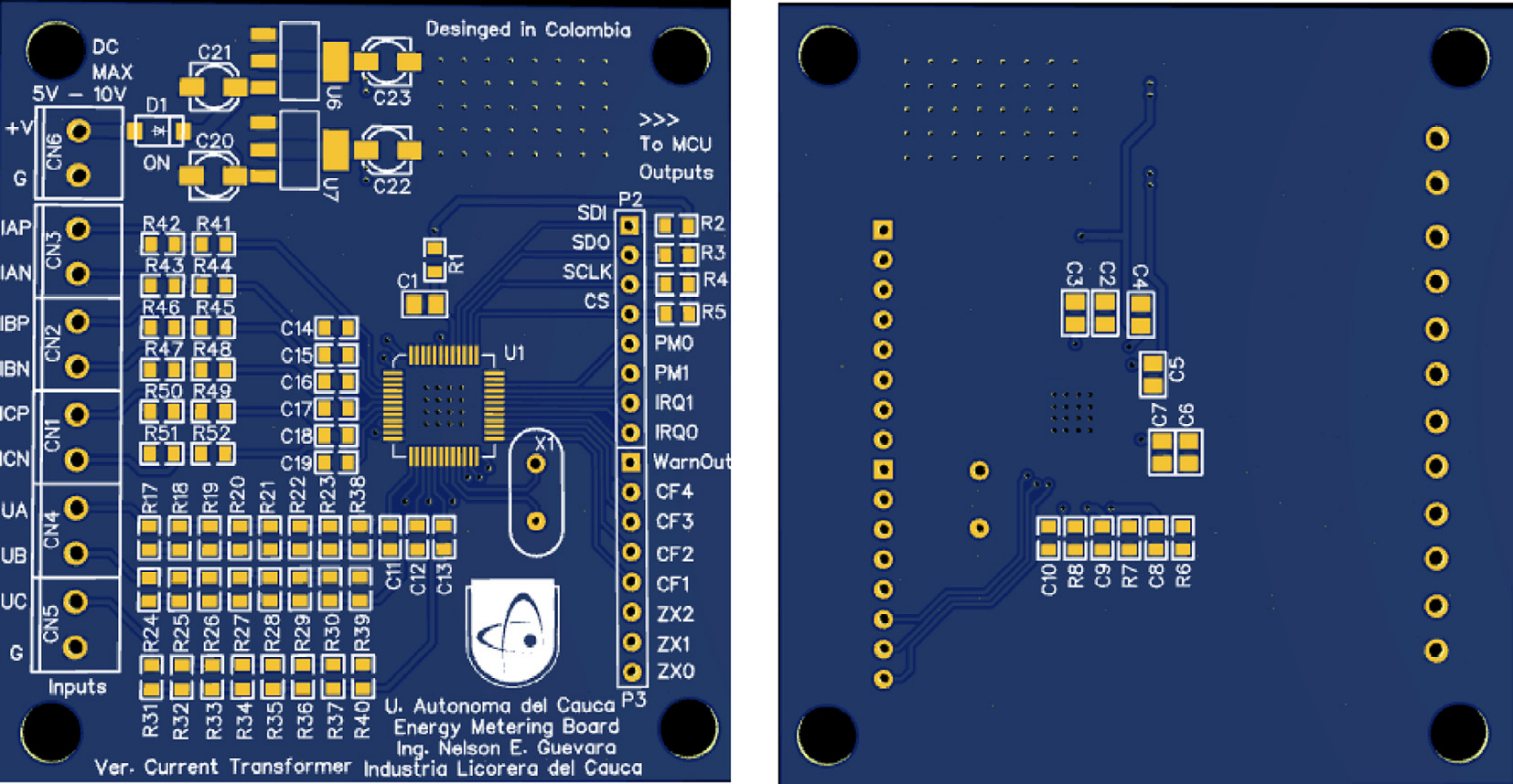
Energy meter PCB diagram made with EasyEda.

For the PCB (Printed Circuit Board) design, EasyEDA online was used, resulting in a reduced size board (101.4 mm × 72.82 mm), Fig. 3 shows the result and the distribution of the elements on it.

**Fig. 4:**
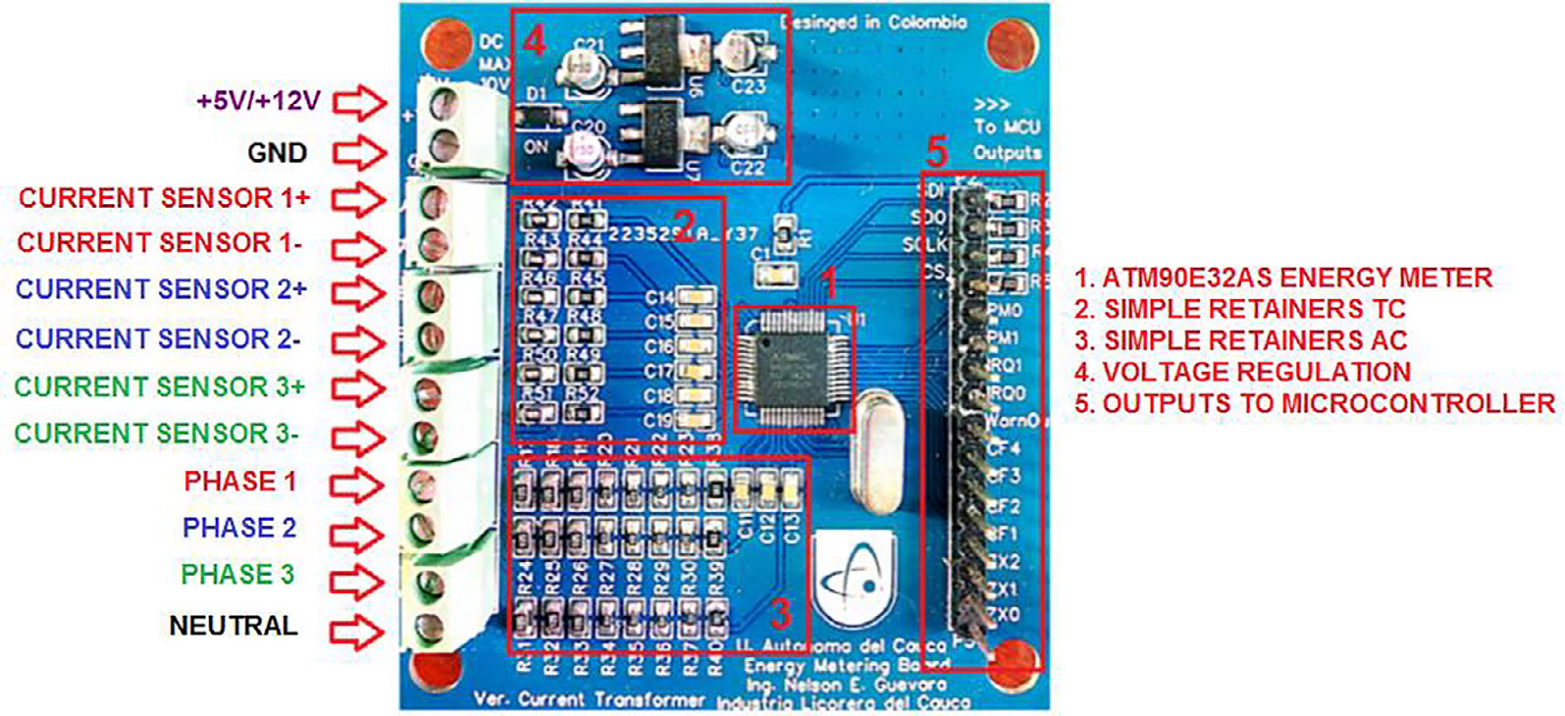
Hardware development result made from a specialized PCBA company.

**Fig. 5:**
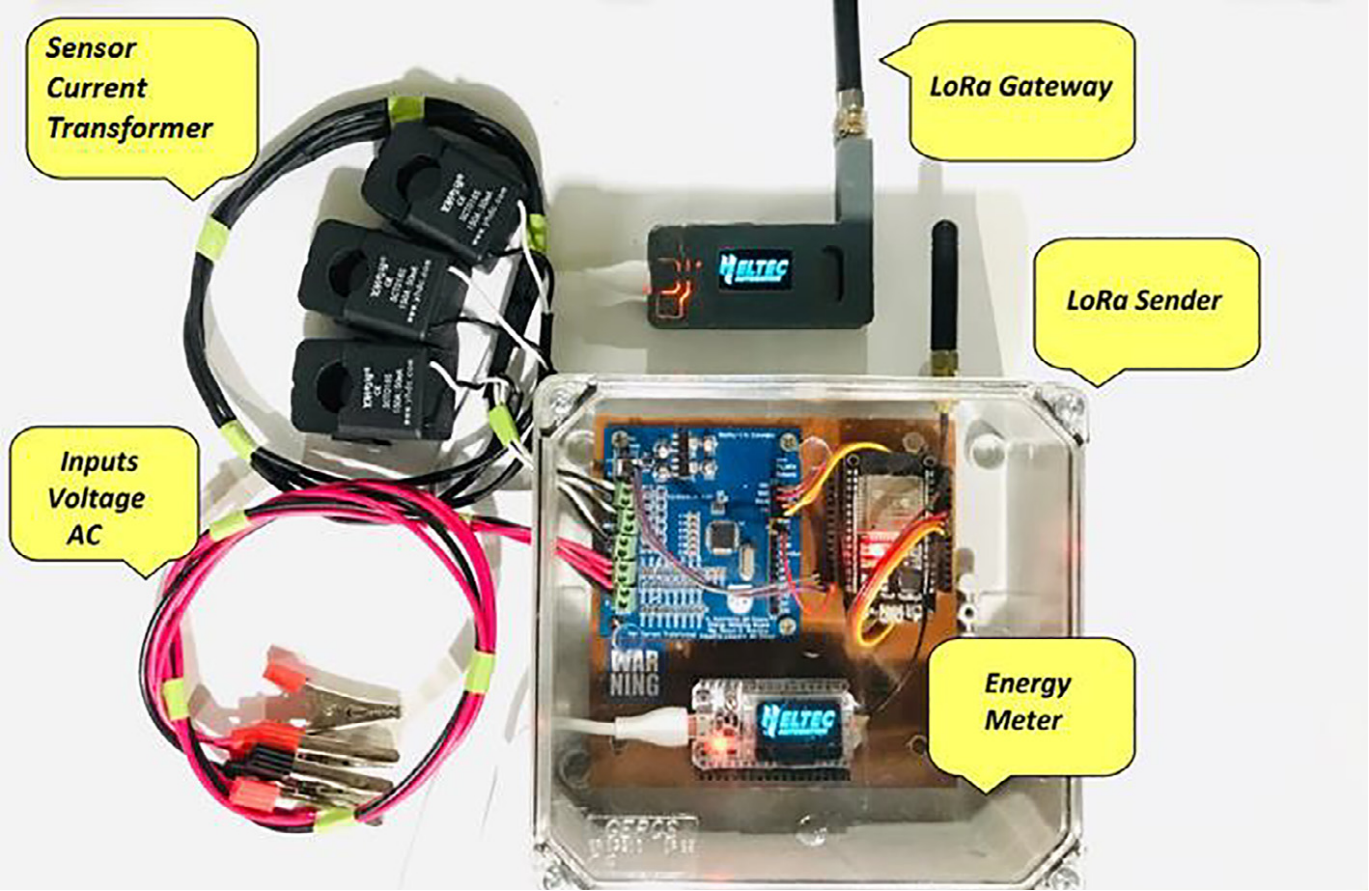
Hardware development result.

**Fig. 6:**
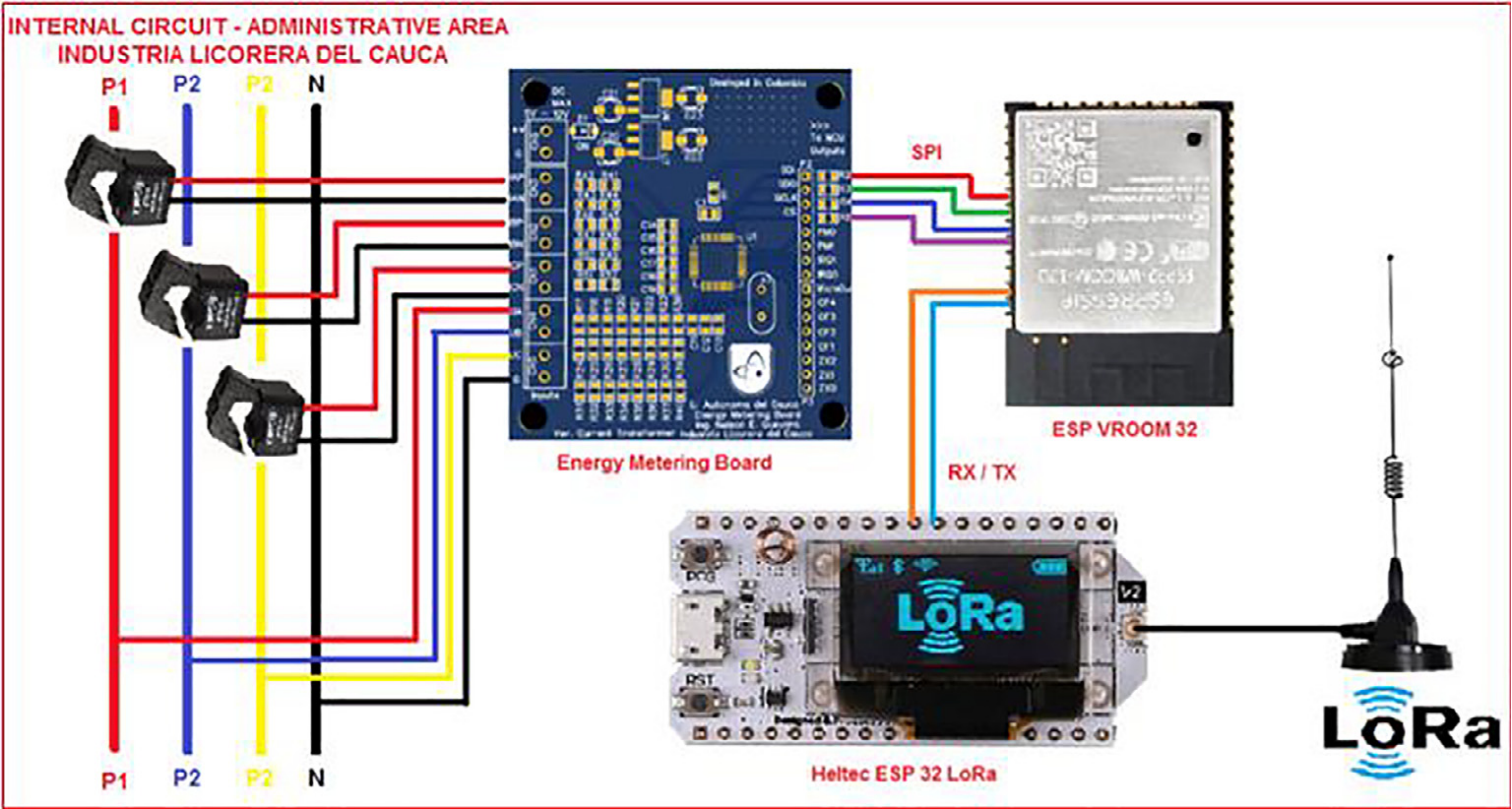
General connection diagram for IoT meter system.

**Fig. 7:**
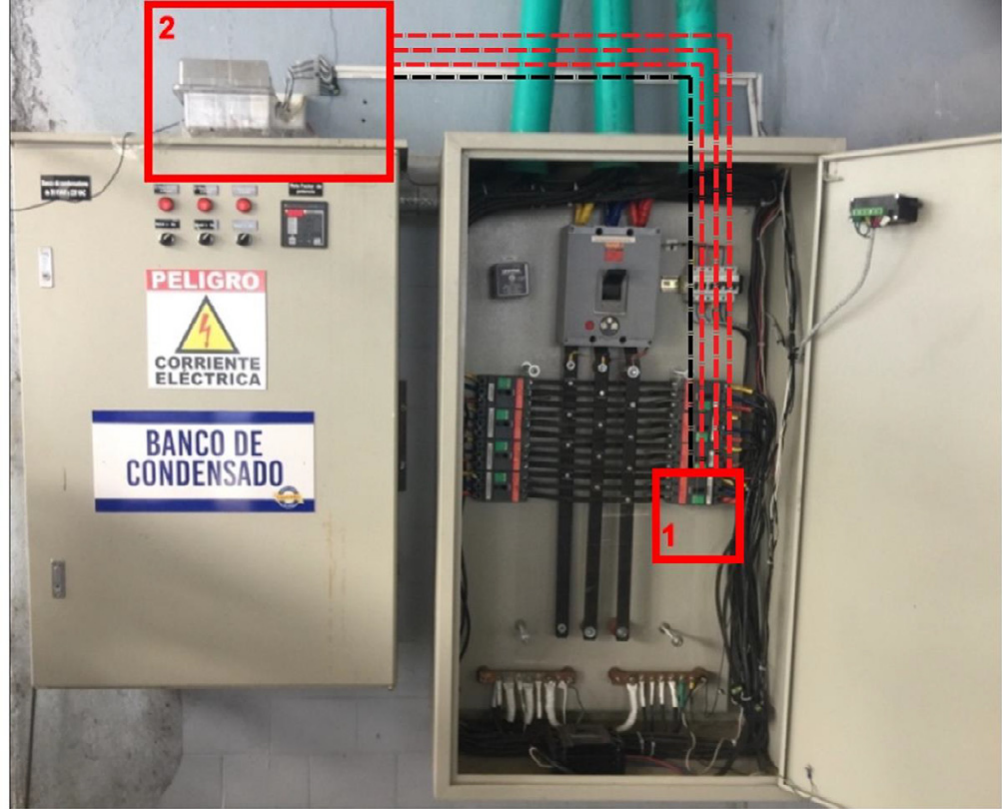
Connection made in administrative area circuit.

The most relevant features of the electronic system are listed below:

• It allows the measurement of current, voltage, active power, reactive power, apparent power, power factor, network frequency, important variables for the analysis of consumption and quality of electrical energy for up to three phases.
• It is an easy to install system.
• It is a portable system.
• It allows to transmit the information of the variables from the measurement point to a maximum distance of 5 Km in line of sight.

## Design files

The following table shows the figures that correspond to the design of the proposed IEMS.

**Table:** 

**Design file name**	**File type**	**Open Source license**	**Location of the fie**
System architecture	Figure (PNG)	CC BY 4.0	Included in the article (Fig. 1)
Schematic diagram reference for energy meter	Figure (PNG)	CC BY 4.0	Included in the article (Fig. 2)
Energy meter PCB diagram made with EasyEda	Figure (PNG)	CC BY 4.0	Included in the article (Fig. 3)
Hardware development result made from a specialized PCBA company	Figure (PNG)	CC BY 4.0	Included in the article (Fig. 4)
Hardware development result	Figure (PNG)	CC BY 4.0	Included in the article (Fig. 5)
General connection diagram for IoT meter system	Figure (PNG)	CC BY 4.0	Included in the article (Fig. 6)
Connection made in administrative area circuit	Figure (PNG)	CC BY 4.0	Included in the article (Fig. 7)
Reception data - LoRa gateway	Figure (PNG)	CC BY 4.0	Included in the article (Fig. 9)
Web application front end developed with HTML, CCS and JavaScript	Figure (PNG)	CC BY 4.0	Included in the article (Fig. 10)
Graphic section	Figure (PNG)	CC BY 4.0	Included in the article (Fig. 11)
Styles of graphics and reports	Figure (PNG)	CC BY 4.0	Included in the article (Fig. 12)

The repository http://dx.doi.org/10.17632/bkhrks6x3m.2 contains the files needed to build the IEMS. These files are classified in folders as follows:

• *Board_Production_Files* folder: Contains Gerber files and bill of materials (BOM), which are necessary for manufacturers to produce the electronic board.
• *ESP32MCU_EnergyMeter_Firmware* folder: Contains a source code developed in Arduino IDE used to program the ESP32 VROOM.
• *ESP32LoRa_Sender_Firmware* folder: Contains a source code developed in Arduino IDE used to program the Heltec ESP32 LoRa on the energy meter.
• *ESP32LoRa_Gateway_Firmware* folder: Contains a source code developed in Arduino IDE used to program the Heltec ESP32 LoRa as gateway.
• *Part For Enclosute_CAD_Files* folder: Contains the Solid Edge files to make the protection case for the gateway.

## Bill of materials

The list of materials used in the design of the IEMS are presented in the following table.

**Table:** 

**Designator**	**Component**	**Number**	**Cost per unit - currency**	**Total cost - currency**	**Source of materials**	**Material type**
Espressif	ESP32 VROOM 160 MHz	1	$ 8.8 USD	$ 8.8 USD	Electrotekmega	Other
Heltec	WiFi LoRa 32 (V2.1) 915 MHz	2	$ 36.69 USD	$ 73.38 USD	Electrotekmega	Other
Current Transducers	YHDC SCT016S Split Core 150A/50 mA	3	$ 12.11 USD	$ 36.33 USD	Electrotekmega	Other
Polyphase Measurement Integrated Circuit	Atmel M90E32AS	1	$ 3.08 USD	$ 3.08 USD	Digikey	Other
Crystal	16.384 MHz	1	$ 0.66 USD	$ 0.66 USD	Digikey	Other
Resistor	1KΩ C0805 SMD	12	$ 0.088 USD	$ 1.05 USD	Digikey	Other
Resistor	10KΩ C0805 SMD	5	$ 0.088 USD	$ 0.44 USD	Digikey	Other
Resistor	240KΩ C0805 SMD	21	$ 0.088 USD	$ 1.84 USD	Digikey	Other
Resistor	2.4 Ω C0805 SMD	6	$ 0.088 USD	$ 0.52 USD	Digikey	Other
Capacitor	100nF C0805 SMD	5	$ 0.027 USD	$ 0.135 USD	Digikey	Ceramic
Capacitor	10uF C1206 SMD	1	$ 0.027 USD	$ 0.027 USD	Digikey	Ceramic
Capacitor	4.7uF C1206 SMD	1	$ 0.027 USD	$ 0.027 USD	Digikey	Ceramic
Capacitor	18nF C0805 SMD	12	$ 0.027 USD	$ 0.324 USD	Digikey	Ceramic
Capacitor	ELECTRO-SMD 3.8 mm	4	$ 0.26 USD	$ 1.04 USD	Digikey	Other
Diode	DO-214AC SMD	1	$ 0.23 USD	$ 0.23 USD	Digikey	Other
Regulator	LD1117AS33TR-SOT-223 (3.3 V)	2	$ 0.47 USD	$ 0.94 USD	Digikey	Other
Connectors	MKDS1/2–3.81	6	$ 0.025 USD	$ 0.15 USD	Digikey	Other
Connectors	Header-Male-2.54_1x8	2	$ 0.015 USD	$ 0.03 USD	Electrotekmega	Other

For the construction of the device that conforms the IEMS, different electronic components are needed that can be acquired in electronics stores such as Digikey, Arrow, or Mouser, also it will be necessary the use of CAD files for the fabrication of plastic housings. All the information can be found in the Board_Production_File folder of the repository presented in the previous section.

## Build instructions

To manufacture the energy meter that conforms the IEMS, specialized services of design and manufacture of electronic systems are needed. Companies that own equipment needed to perform the mentioned processes, specifically require a detailed number of gerber and BOM files, which clearly describe the characteristics of the PCB (Board_Production_Files.zip), to subsequently deliver to the user an electronic prototype like the one presented in Fig. 4.

• Once you have an electronic board and the electronic components assembled on the main board, the first step is to load the firmware files into the ESP32 control unit (ESP32MCU_EnergyMeter_Firmware.rar) and Sender LoRa (ESP32LoRa_Sender_Firmware.rar). This task requires the Arduino IDE application.
• The second step is to load the firmware file for the LoRa Gateway (ESP32LoRa_Gateway_Firmware_Firmware.rar). This task requires the Arduino IDE application.

Fig. 5, shows the energy meter, the gateway and other components that make up the IEMS.

## Operation instructions

The IEMS is designed to be connected to a distribution circuit, it is recommended to install in a distribution box, at no more than one meter from the connection bars, avoid placing near equipment that generates electromagnetic noise such as motors, to ensure wireless communication it is recommended that the device is not completely enclosed in a metallic box this generates faraday cage effect that would damage the transmission by the LoRa network. The connections for voltage sensing go directly to the busbars or terminals of the circuit to be measured, the current transformers being non-invasive must be installed as clamps on each of the phases to be sensed. Fig. 6 shows the general connection diagram and Fig. 7 shows an installation example.

**Fig. 8:**
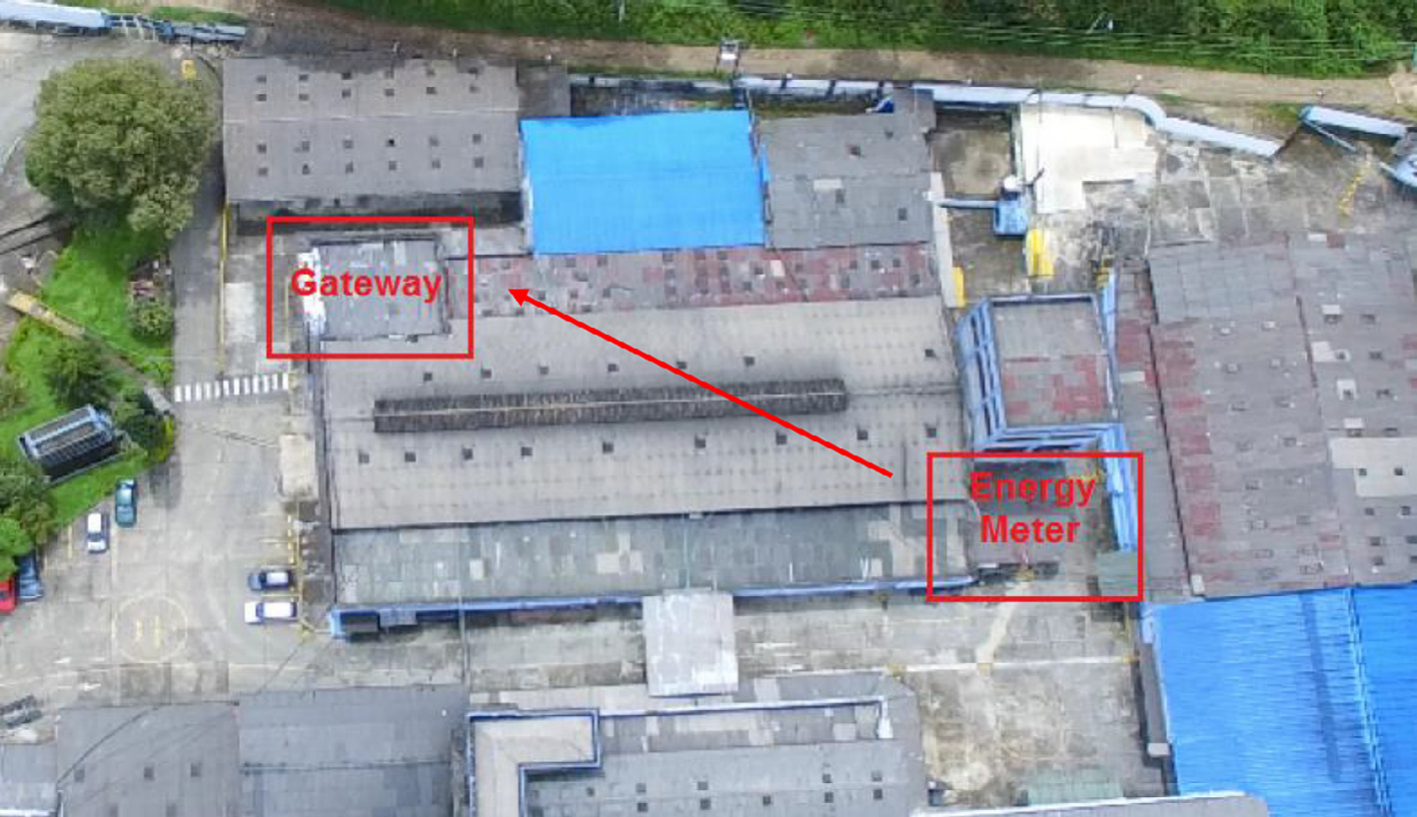
Installation of devices for case of study monitoring, aerial view.
• For the connection in the firmware must be supplied the access information to the dome network are SSID and password once the firmware is loaded check connection to Wi-Fi network and reception of electrical data in the LoRa gateway module (Fig. 9).

• Red box 1: Connection to test circuit output.
• Red box 2: Location of IEMS.
• Once the connections have been made, the IEMS power supply must be energized using a 110VAC to 5V 2A power adapter.
• The gateway must be located and powered with a 110V to 5V power supply adapter at a distance with coverage. Fig. 8 shows the example of the case study, where the distance between the two devices is approximately 800 m.

**Fig. 9:**
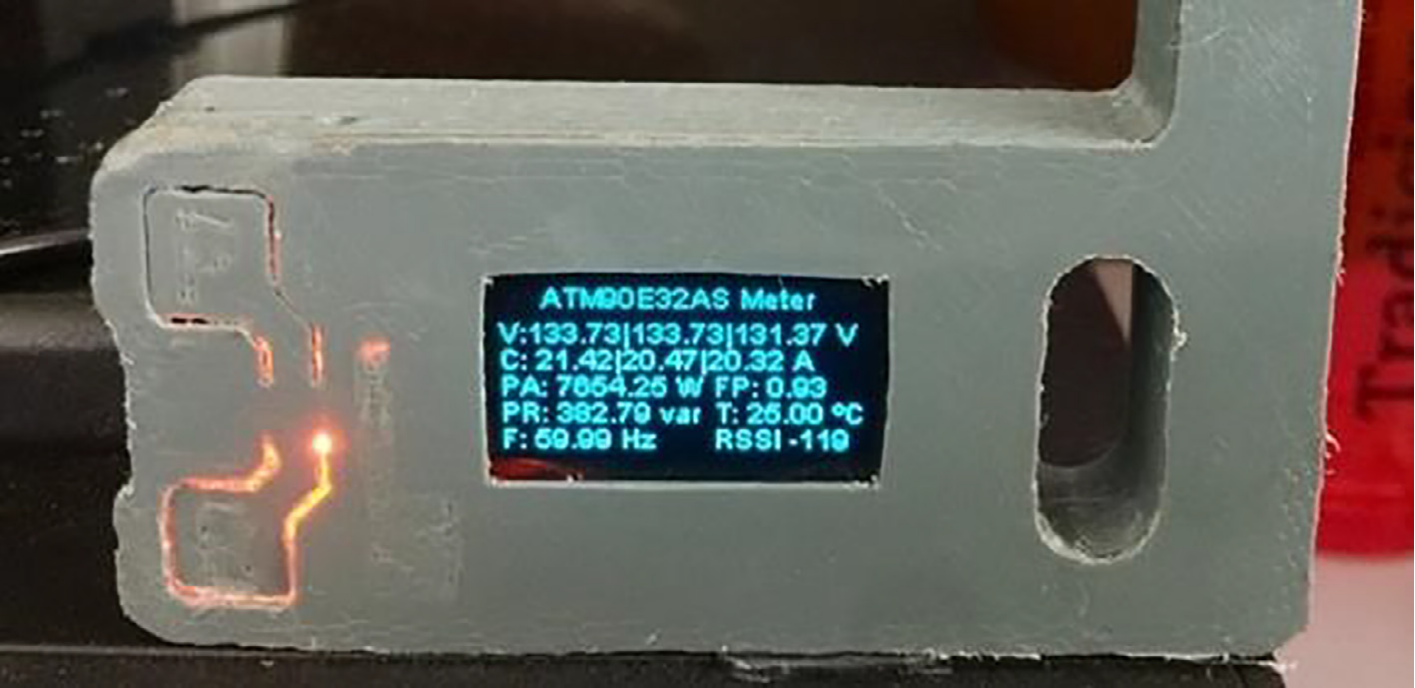
Reception data – LoRa gateway. (Own source).
• After installing the meter and the LoRa gateway and these are turned on, the next step is to install the web application (Fig. 10) included in the Web_Application_Files folder of the repository, this application could be installed as a local mode or uploaded to a server (instructions are detailed in the software folder in the repository), where the electrical data collected by the system will be stored periodically.

**Fig. 10:**
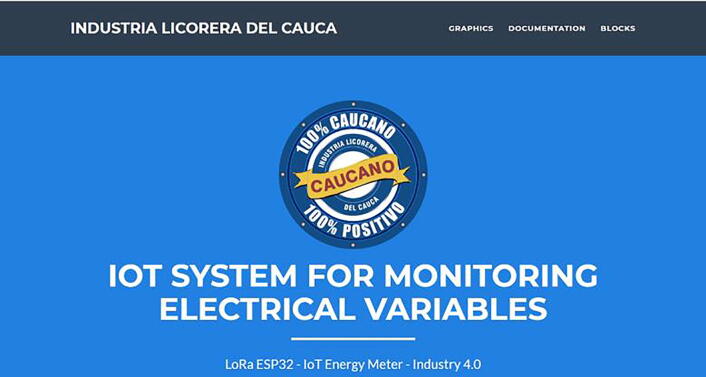
Web application front end developed with HTML, CCS and JavaScript. (Own source).
• Once the web application has been executed, select an electrical variable to observe the data collected from it (Fig. 11).

**Fig. 11:**
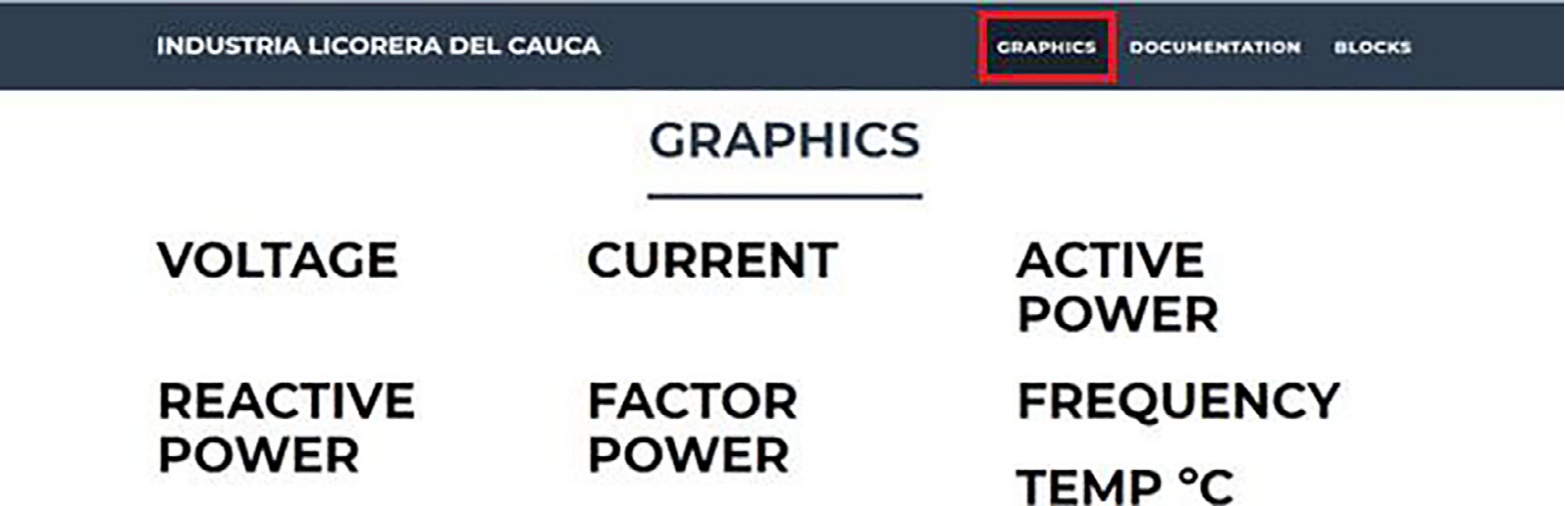
Graphics section. (Own source).
• The web application will offer the user different graphical reports such as Bars, Meters or Microsoft Excel compatible downloadable files (Fig. 12).

**Fig. 12:**
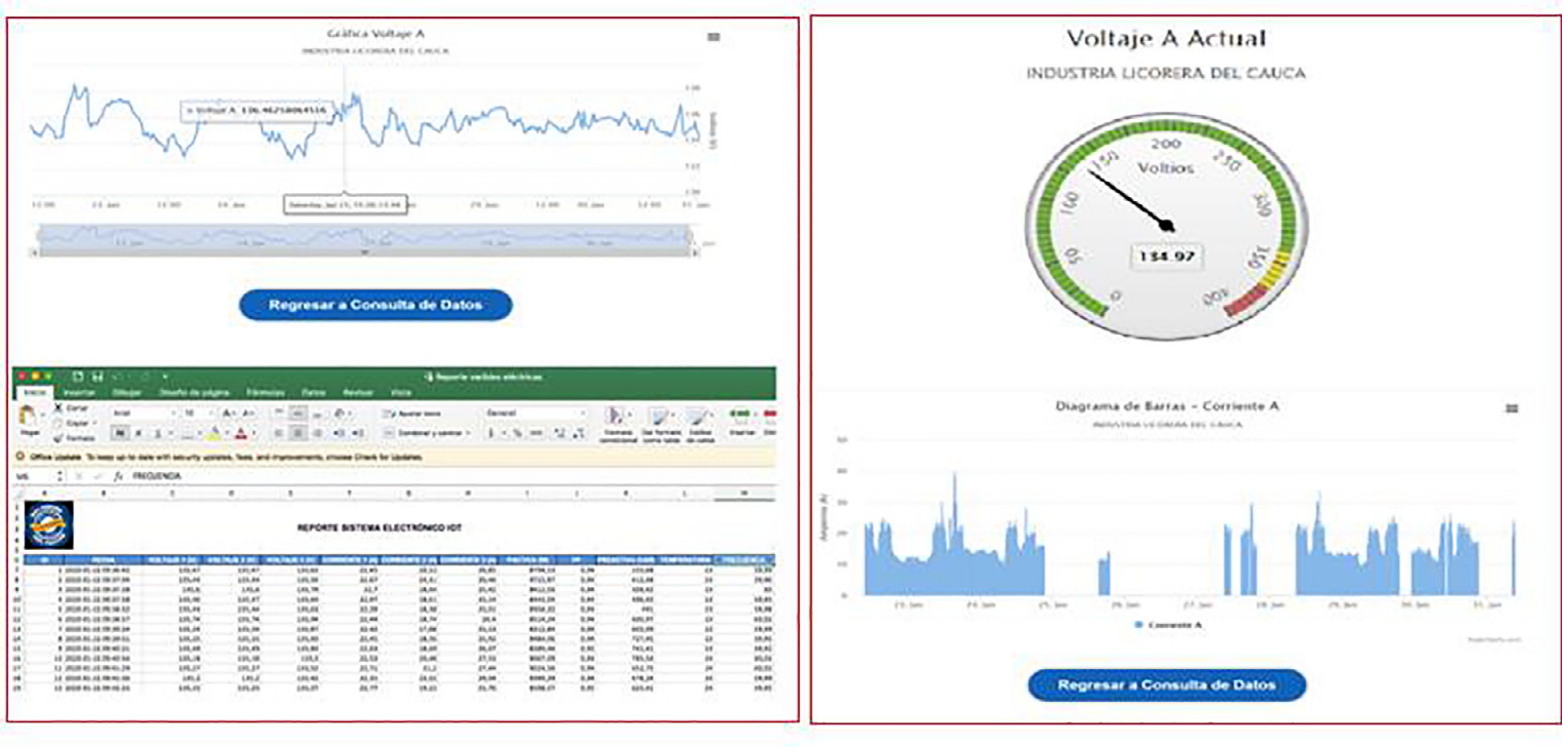
Styles of graphics and reports. (Own source).

## Validation and characterization

**Fig. 13:**
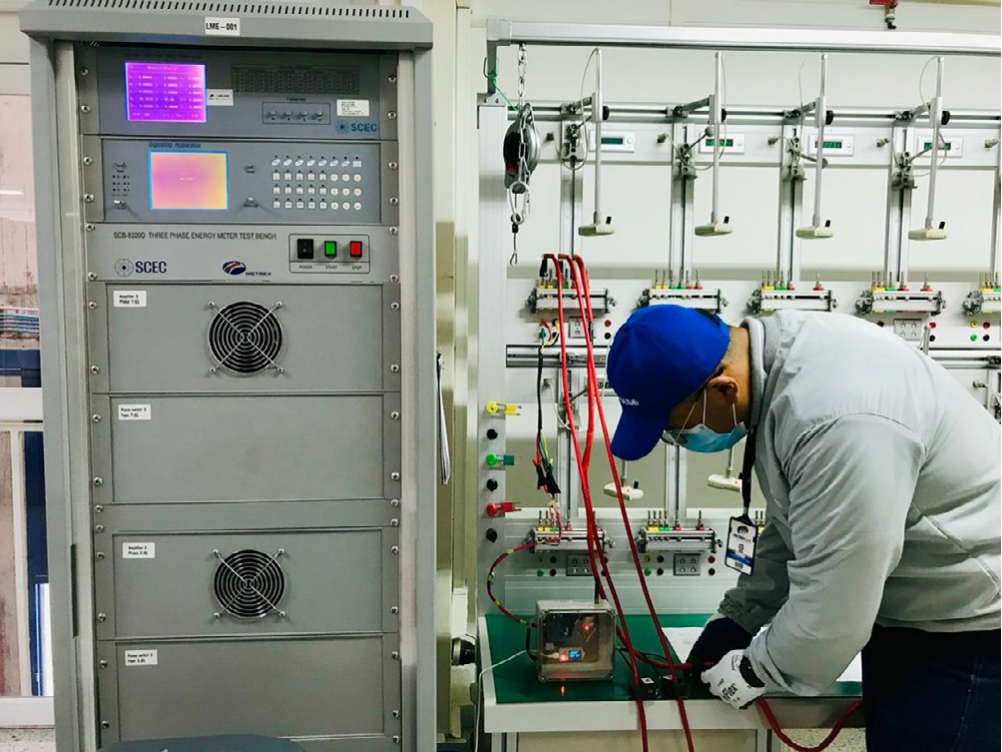
Validation of IEMS in laboratory calibration bench.

During the validation phase of the prototype, a contrast test was performed on a specialized bench of an accredited meter calibration laboratory belonging to the Colombian company Metrex S.A, as shown in Fig. 13. The values obtained are presented in Table 1, Table 2.

**Table 1:** Current comparison between Metrex Meter test bench and IEMS.

Current Laboratory bench [A]	Current IF1 [A]	**Relative error IF1 %**	Current IF2 [A]	**Relative error IF2 %**	Current IF3 [A]	**Relative error IF3 %**
1	0.93	**7.0**	0.93	**7.0**	0.93	**7.0**
2	1.87	**6.5**	1.88	**6.0**	1.87	**6.5**
5	4.73	**5.4**	4.73	**5.4**	4.73	**5.4**
10	9.47	**5.3**	9.47	**5.3**	9.47	**5.3**
20	19.0	**5.0**	19.0	**5.0**	19.0	**5.0**
30	28.7	**4.3**	28.7	**4.3**	28.8	**4.0**
40	38.0	**5.0**	38.0	**5.0**	38.0	**5.0**
50	47.5	**5.0**	47.6	**4.8**	47.6	**4.8**
60	56.96	**5.0**	57.2	**4.6**	57.2	**4.6**

**Table 2:** Voltage comparison between Metrex Meter test bench and IEMS.

Voltage Laboratory bench [V]	Voltage VF1 [V]	**Relative error VF1 %**	Voltage VF2 [V]	**Relative error VF2 %**	Voltage IF3 [V]	**Voltage error VF3 %**
100	99.8	**0.2**	100.9	**0.9**	100.5	**0.5**
110	109.9	**0.09**	110.8	**0.72**	110.5	**0.45**
120	119.9	**0.08**	120.5	**0.41**	120.5	**0.41**
130	129.9	**0.07**	131.0	**0.76**	130.6	**0.46**

According to the data presented in Table 1, Table 2, it is concluded that the percentage errors are low 0.9% at most for the voltage variable and up to 7% for current, however it should be noted that this error can be reduced by applying an adjustment protocol for which equations supplied by the manufacturer of the M90E32AS chip can be used [12], given the purpose of the project, with the obtained values the equipment can be used as a diagnostic tool but not for billing or certification processes where greater accuracy is required and for which a second process for laboratory adjustment will be required.

To evaluate the IEMS, it was installed in the main electrical cabinet of a local company, specifically in the circuit of the administrative area. The connection of the phases to be measured was done through an easily accessible terminal block for safety reasons. During the evaluation the device captured 16,857 samples during five days, with a web server update rate of 30s. The data were stored in a database created with MySQL, the following electrical variables were captured:

• Voltage over all electric phases, VF1, VF2, VF3.
• Current over all electric phases, IF1, IF2, IF3.
• Active power
• Reactive power
• Power factor
• Frequency
• Temperature

The following graphs were produced by the web application, in which a cut of one day was made to analyze the behavior of the electric variables in the circuit under monitoring.

**Fig. 14:**
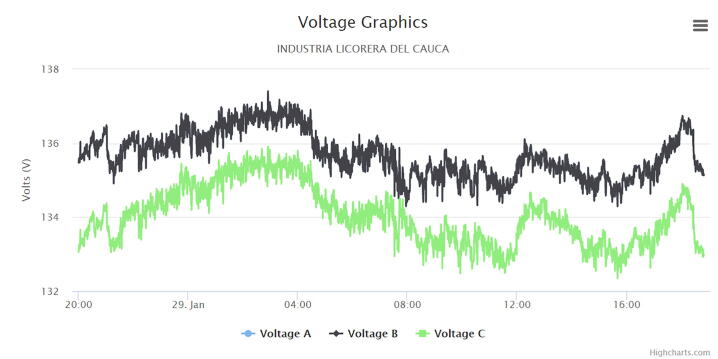
Voltage graph. (Own source).

Fig. 14 presents the voltage in the three phases VF1, VF2 and VF3 where the following behaviors were observed:

• According to Fig. 14, it can be observed that from 8AM to 4PM, there is a slight decrease in the voltage of the phases, in this space of time it is known that there is a full development of almost all the administrative work of the company (secretarial work, office staff, reception staff, among others).

**Fig. 15:**
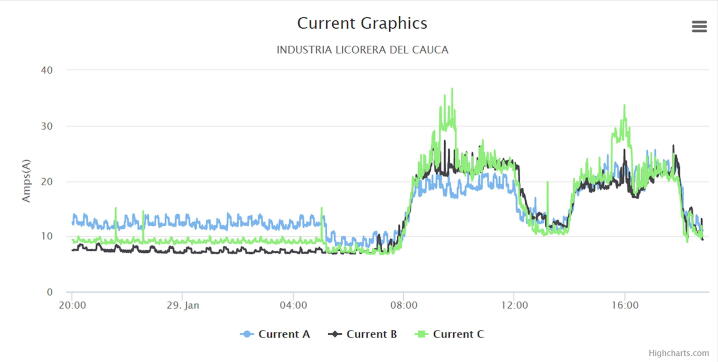
Current graph. (Own source).

Fig. 15 shows the current data in the three phases of the circuit under monitoring called IF1, IF2, IF3, this is one of the most important variables from which valuable information can be extracted, the following conclusions are drawn from the graphical analysis.

• Current consumption increases during working hours from 8AM to 12PM and from 2PM to 6PM.
• There is a significant consumption of 10A at night, equivalent to the operation of two web server cabinets and air conditioners used for cooling.
• The average consumption in each phase is 20A.
• There is a decrease in current between 12PM and 2PM, which does not reach 10A per phase, due to the fact that equipment such as cell phones, computers and others are probably connected during lunch hours.
• After 2PM there is an increase in current and it is in phase 1 (Blue) where a maximum peak of 36 amps occurs around 3:30PM, which in consultation with the staff and according to habits is highly likely to be due to the ignition of electric stoves to prepare coffee and air conditioners.

**Fig. 16:**
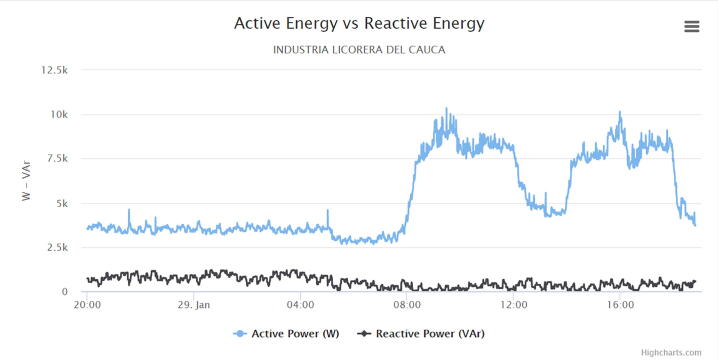
Active energy and reactive energy graph. (Own source).

Fig. 16 shows the active and reactive energy delivered by the system in the three phases. The active energy (Blue) is associated with all the electrical work required in the circuit, the observed behaviors for this variable are as follows:

• The average value of the active energy of the three phases during non-working hours is 4KW and 13KW maximum during working hours.
• The active energy allows the user to know the power required in the circuit of the administrative area, with the purpose of making a load reduction or balancing plan in the circuit.

In relation to reactive energy, which must maintain permissible values, it can be concluded:

• The average value in non-working hours corresponds to a value of 250VR and 1100VR in working hours.
• The values of this variable are within the permitted levels, which does not exceed the active energy by more than 50%, in accordance with Resolution 065 of 2012 by the Energy and Gas Regulation Commission by the CREG [16].
• The values detected by the IEMS correspond to the fact that there are no motors or large machines that require magnetic field, but there are variations due to the switching process of some regulated source circuits connected to the network.

**Fig. 17:**
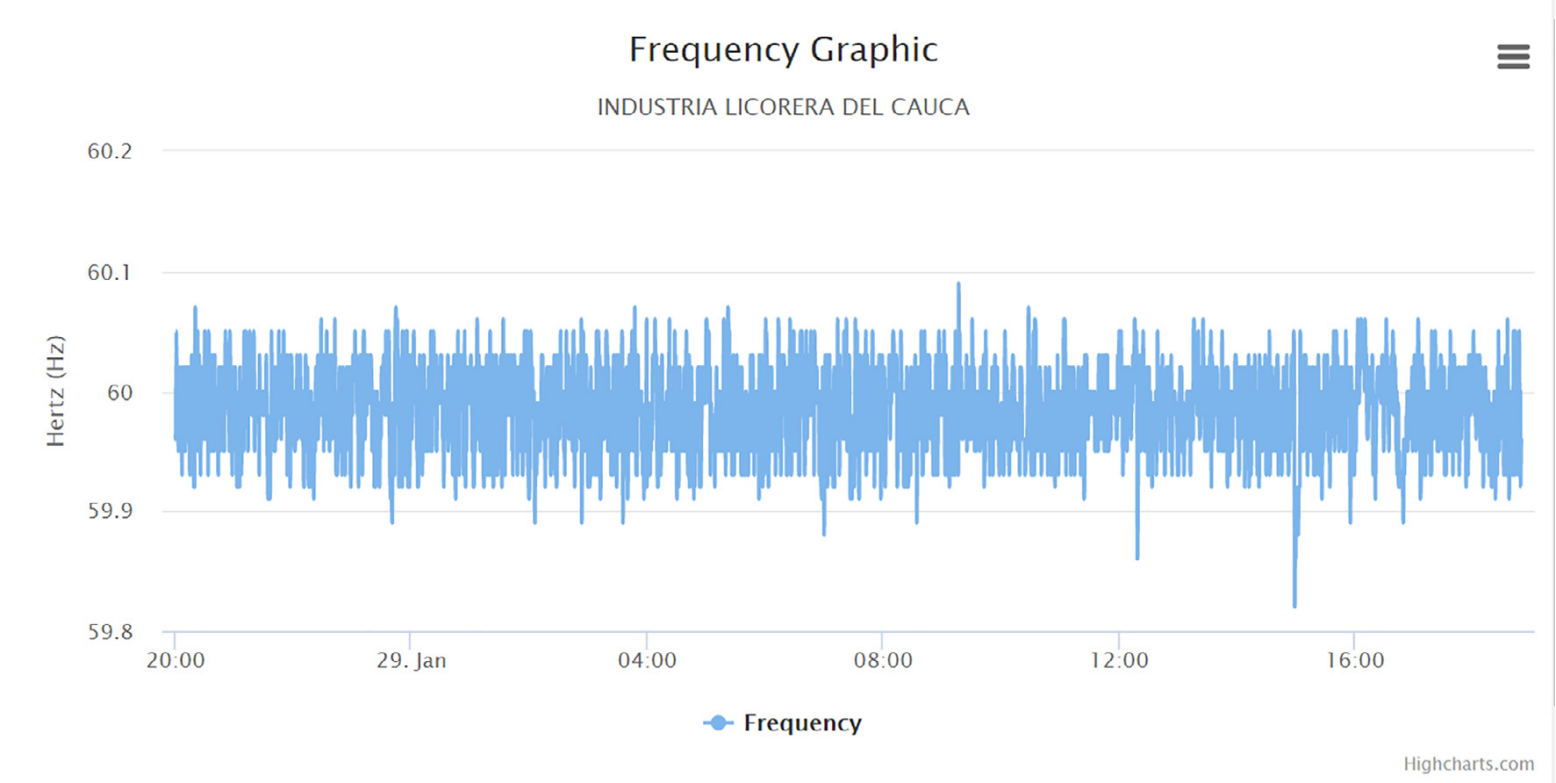
Frequency graph. (Own source).

Fig. 17 shows the frequency graph of the phases sampled by the system, in the graph it is evident that the frequency is varying around 60 Hz, this indicates that there is no marked harmonic distortion, which obeys the resolution 025 of 1995 by the CREG [17], which dictates that the range of variation should be between 59.89 and 60.2 Hz.

**Fig. 18:**
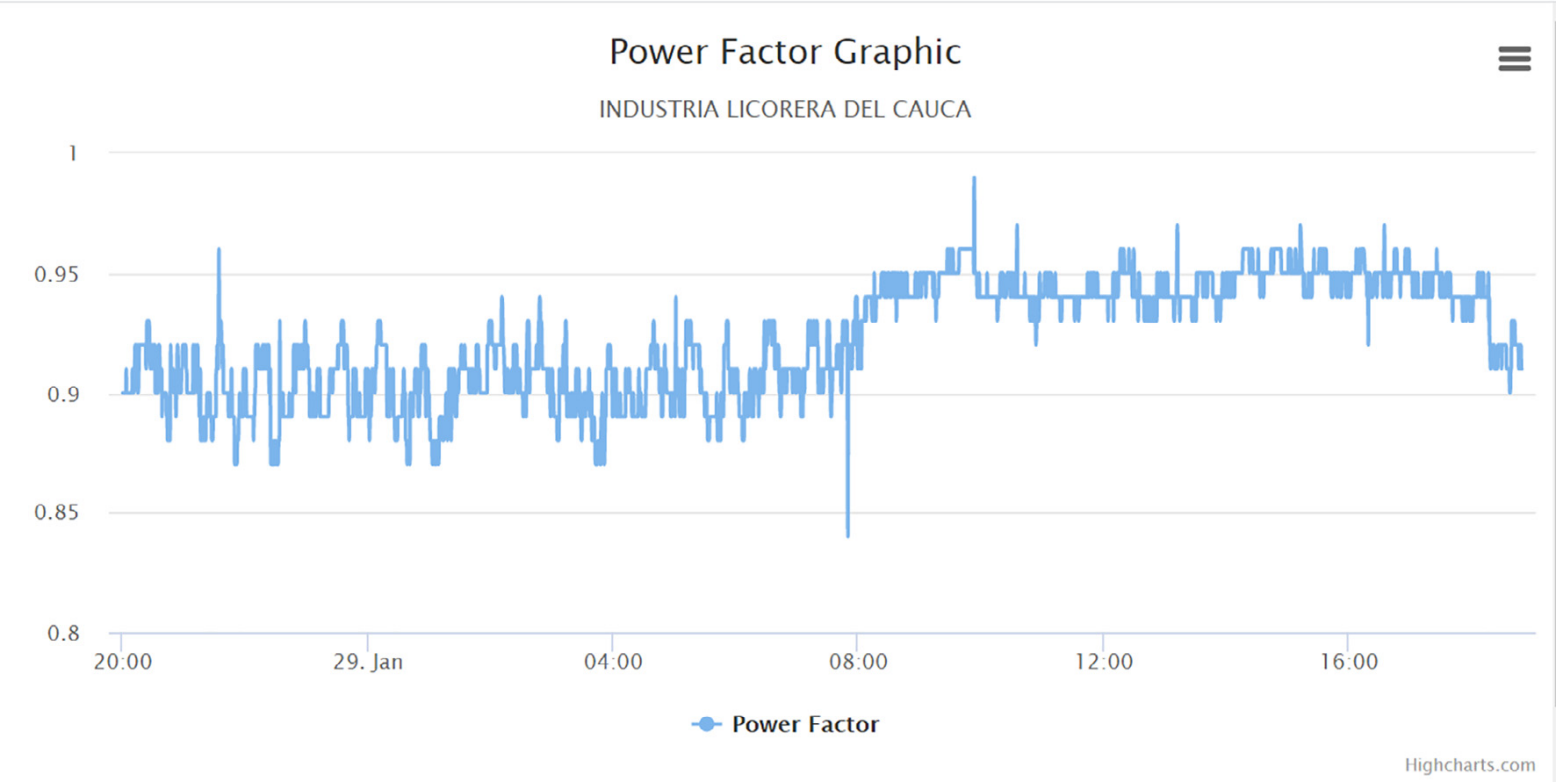
Power factor graph. (Own source).

Fig. 18 shows the behavior of the total power factor of the phases in the sampling period. The observed behaviors for the power factor are as follows:

The average variation of this variable is 0.09, maintaining a value very close to 1 between 0.90 and 0.99, which is within the permitted levels, obeying article 25 of resolution 108 of 1997 by the CREG [18], which is the entity that regulates the admissible power factor for companies in Colombia.

**Fig. 19:**
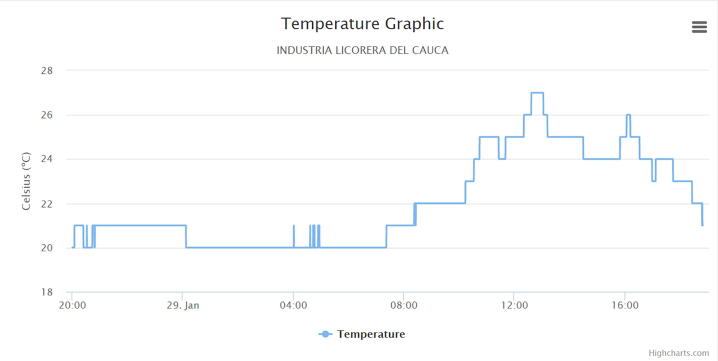
Temperature graph. (Own source).

Fig. 19 shows the temperature captured by the energy meter. Since the meter is located very close to the main electrical cabinet, the temperature is an important parameter to associate the data with ambient temperature values. The temperature peak corresponds to the system operation and the effect of the mid-day temperature increase. Additionally, it is important to note that if this device is located in an area where there are air conditioners, the temperature and energy consumption can be contrasted.

## Discussion

The study associated with the development of the device proposed a low-cost IoT electronic system for the estimation and visualization of energy consumption in low voltage electrical circuits. During the evaluation phase, described in Validation and characterization section, it is proposed that it is possible to estimate electrical variables such as: voltage, current, reactive energy, active energy, power factor, among others; for a diagnostic use with low-cost equipment such as the IEMS. Consequently, the results provided by the IoT system made it possible to identify load unbalances between phases, base consumption, excessive consumption, and power quality in a circuit of the case study company (Fig. 15). The diagnosis allowed the development of energy management plans and the identification of obsolete equipment with high energy consumption for their replacement, such as electric stoves, refrigerators, and air conditioners.

Therefore, it is concluded that the proposed IoT system is a viable alternative for the estimation of energy consumption due to its low cost, easy installation, replicability and finally that it does not require licenses for operation. Additionally, it is important to highlight that having this type of electronic systems, for use as diagnostic tools especially when resources are limited and the use of professional measuring instruments for obtaining consumption profiles in small factories or homes can be avoided.

## Limitations

The test circuit used for the M90E32AS variable meter chip (Fig. 3) was developed based on test application notes, however, it should be improved by adding robust high voltage protections; therefore, future work will focus on designing an appropriate protection stage for variable acquisition. Moreover, to increase the portability of the prototype, the local web server and web application will be installed and run on a SCB (Single Computer Board) such as a Raspberry Pi.

## Conclusions

According to the tests developed in the factory, the proposed low-cost IEMS becomes a promising hardware tool particularly in small factories or homes to estimate and monitor electrical variables such as voltage and current with a percentage error of 0.9% and 7%. Additionally, the data captured with the IEMS and the graphic option allowed to analyze the behavior of electrical variables such as: voltage, current, active energy, reactive energy, power factor and frequency to contrast them with the values allowed by the regulatory body in the country. Finally, through this analysis, energy management plans were developed to reduce energy expenditure within the company demonstrating its usefulness demonstrating its usefulness.

**Human and animal rights**.

For the implementation of the IEMS, it was necessary to count on the collaboration of qualified personnel from the electrical area of the company who, with their verbal consent, agreed to make the high voltage connections in the electrical distribution boards. The activities developed have contraventions against human rights or animal rights.

## Declaration of Competing Interest

The authors declare that they have no known competing financial interests or personal relationships that could have appeared to influence the work reported in this paper.
